# Diagnostic performance of diffusion-weighted magnetic resonance imaging in assessing lymph node metastasis of esophageal cancer compared with PET

**DOI:** 10.1007/s10388-019-00704-w

**Published:** 2019-12-09

**Authors:** Kiyohiko Shuto, Tsuguaki Kono, Toru Shiratori, Yasunori Akutsu, Masaya Uesato, Mikito Mori, Kazuo Narushima, Shunsuke Imanishi, Yoshihiro Nabeya, Noriyuki Yanagawa, Shinichi Okazumi, Keiji Koda, Hisahiro Matsubara

**Affiliations:** 1 Department of Frontier Surgery, Chiba University Graduate School of Medicine, Chiba, Japan; 2 Department of Surgery, Teikyo University Chiba Medical Center, Anesaki, 3426-3, Ichihara, Chiba 299-0111 Japan; 3 Department of Surgery, Toho University Sakura Medical Center, Sakura, Chiba Japan; 4 Department of Radiology, Chiba University Graduate School of Medicine, Chiba, Japan

**Keywords:** Esophageal cancer, Diffusion-weighted MRI, PET, Lymph node

## Abstract

**Background:**

Although diffusion-weighted magnetic resonance imaging (DWI) for detecting lymph node (LN) metastasis is reported to be a successful modality for primary malignant tumors, there are few studies relating to esophageal cancer. This study aimed to clarify the diagnostic performance of DWI for assessing LN metastasis compared with positron emission tomography (PET) in patients with esophageal squamous cell cancer (eSCC).

**Methods:**

Seventy-six patients with histologically proven eSCC who underwent curative esophagectomy without neoadjuvant treatment were reviewed retrospectively. Harvested LNs were divided into 1229 node stations with 94 metastases. Diagnostic abilities and prognostic significance were compared.

**Results:**

In a station-by-station evaluation, the sensitivity was higher in DWI than PET (67% vs. 32%, *P *< 0.001). DWI showed more than 80% sensitivity for middle- and large-sized cancer nests and large area of cancer nests. The DWI-N0 group had a better 5-year relapse-free survival rate than the DWI-N+ group (78.5% vs. 34.2%, *P* < 0.001), as did the PET-N0 group. DWI-N status was an independent prognostic factor (hazard ratio [HR], 2.642; *P *= 0.048), as was PET-N status (HR 2.481; *P *= 0.033).

**Conclusions:**

DWI, which depends on cancer cell volume followed by elevated intranodal density, is a non-invasive modality and showed higher sensitivity than PET. It has clinical impact in predicting postoperative survival for patients with eSCC alongside its diagnostic ability and has significant performance in clinical practice.

## Introduction

Esophageal squamous cell cancer (eSCC) is a formidable disease that has a higher rate of lymph node (LN) metastasis than other gastrointestinal malignancies [1]. Accurate nodal assessment is critical in determining a proper treatment strategy, because LN metastasis is one of the most powerful predictors of prognosis of eSCC [2, 3]. Neoadjuvant chemotherapy has become the standard treatment for stage II/III eSCC in Japan [4]; however, LN assessment remains insufficient. Currently, computed tomography (CT), endoscopic ultrasonography (EUS), and 18F-fluorodeoxyglucose positron emission tomography (FDG-PET) are commonly-used staging modalities, whereas each have certain limitations in LN metastases detection. CT has difficulties in detecting normal-sized metastatic LNs and in discriminating inflammation in enlarged nodes [5]. EUS and recent EUS elastography have high sensitivity in nodal detection [6, 7]; meanwhile, they have disadvantages in evaluating nodes distal to the esophageal wall and stenotic tumors. Although PET and PET/CT can sensitively reflect metabolic changes in tissue, its sensitivity for metastatic LNs is approximately 30–60%, which seems rather low despite high corresponding specificities of more than 90% [8, 9].

Since diffusion-weighted magnetic resonance imaging (DWI) was introduced into clinical practice, it has been widely applied to many malignant neoplasms [10–12]. DWI is one of the functional imaging techniques, like PET. Its principles are based on the random motion of water molecules in tissue. Previous reports demonstrated its clinical utility for detecting metastatic LNs in many digestive cancers [13–15]. It has also been applied to eSCC [16, 17]; however, there are few reports relating to LN metastasis [18] and its diagnostic performance is controversial compared with PET. The purpose of this study was to clarify the diagnostic performance of DWI for assessing LN metastasis compared with FDG-PET and to evaluate its prognostic significance for the patients of eSCC.

## Materials and methods

### Patient population

From February 2006 to September 2011, 82 consecutive patients with histologically proven eSCC who underwent curative esophagectomy without neoadjuvant treatment were reviewed retrospectively. Six patients who did not undergo both DWI and PET were excluded. In total, 76 patients were included in this study. The median postoperative follow-up duration was 64.9 months (range 1.6–142.9 months). One case (1.3%) died of pneumonia during their postoperative hospital stay. Three cases (3.9%) were lost to follow-up within 5 years postoperatively.

Within 1 month prior to surgery, patients underwent gastrointestinal endoscopy to obtain biopsy specimens, as well as barium contrast radiography and a contrast-enhanced multidetector-row CT scan from the cricoid cartilage to the lower abdomen with a 1.25-mm slice thickness. According to these modalities, tumors were staged clinically by the tumor-node-metastasis (TNM) classification of the International Union Against Cancer (UICC) at the time, and in this study, we translated them into the newest UICC-TNM classification [19]. The locations of LN stations were re-evaluated according to the Japanese Classification of Esophageal Cancer [20]. In three patients with cStage IV disease (3.9%), two patients were diagnosed as cM1/Stage IVB because of supraclavicular LN metastasis and one was cM0/Stage IVA (Table 1).

**Table 1:** Patient characteristics

Characteristics	Total, *n* = 76
Age, years	67 (41‒82)
Gender, *n* (%)	
Male	64 (84.2)
Female	12 (15.8)
Tumor location in the esophagus, *n* (%)	
Upper thoracic	9 (11.8)
Middle thoracic	35 (46.1)
Lower thoracic	30 (39.5)
Abdominal	2 (2.6)
Histological grade, *n* (%)	
G1	10 (13.2)
G2	55 (72.4)
G3	11 (14.5)
Tumor size, mm	31.5 (8‒100)
Lymphadenectomy, *n* (%)	
Thoraco-abdominal	17 (22.4)
Cervico-thoraco-abdominal	59 (77.6)
Clinical T stage, *n* (%)	
T1	38 (50.0)
T2	14 (18.4)
T3	23 (30.3)
T4a	1 (1.3)
Clinical N stage, *n* (%)	
N0	53 (69.7)
N1	14 (18.4)
N2	8 (10.5)
N3	1 (1.3)
Clinical M stage, *n* (%)	
M0	74 (97.4)
M1	2 (2.6)
Clinical Stage, *n* (%)	
I	35 (46.1)
II	21 (27.6)
III	17 (22.4)
IV	3 (3.9)

Continuous data are shown as median (range)

*LN* lymph node

### Surgical procedures and pathological examination

Patients underwent a complete thoracic esophagectomy with cervico-thoraco-abdominal lymphadenectomy followed by gastric tube reconstruction as standard surgery. Thoraco-abdominal lymphadenectomy was carried out in patients of > 75 years in age or in patients with abdominal esophageal tumors.

LNs were separated from resected esophagus and were assigned specific LN station numbers. Specimens were cut along the long axis, fixed, embedded, and stained with hematoxylin and eosin. In all metastatic LNs, the long axis size of the whole LN and the intranodal cancer nest size were measured. Additionally, the cancer nest occupying an area of the whole node was categorized as follows: small area, less than one-third; middle area, less than two-thirds; and large area, more than or equal to two-thirds. If two or more metastatic LNs were involved in one nodal station, the largest node was used for evaluation.

### MR imaging and analysis

**Fig. 1:**
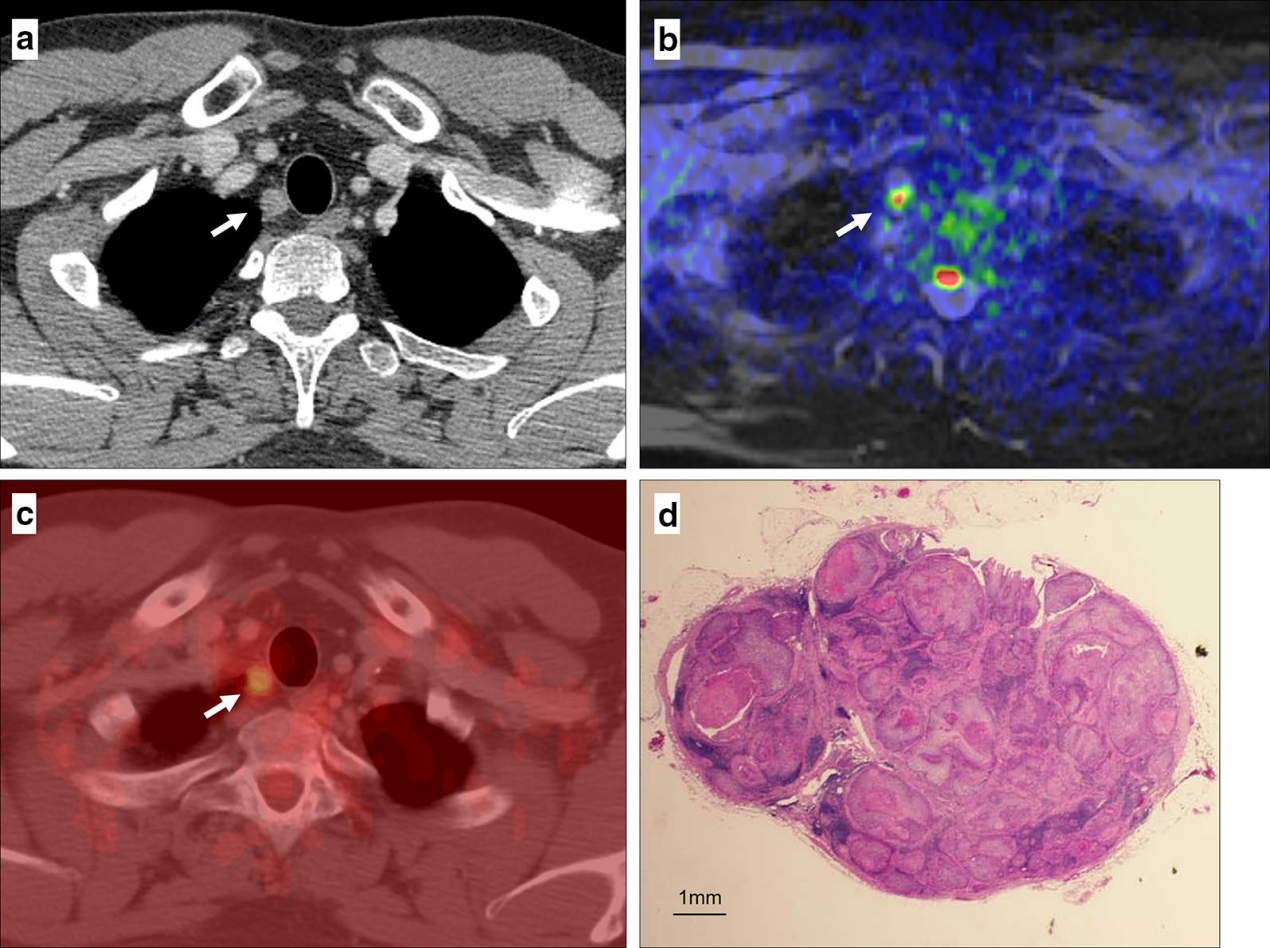
True positive lymph node of 10 mm in size by DWI and PET (arrow). **a** Thoracic paratracheal lymph node was detected on contrast-enhanced CT as metastasis. It was hyperintense on DWI (**b**) and hyperaccumulate on PET (**c**). **d** The node was identified as metastatic with 100% of intranodal cancer nest

**Fig. 2:**
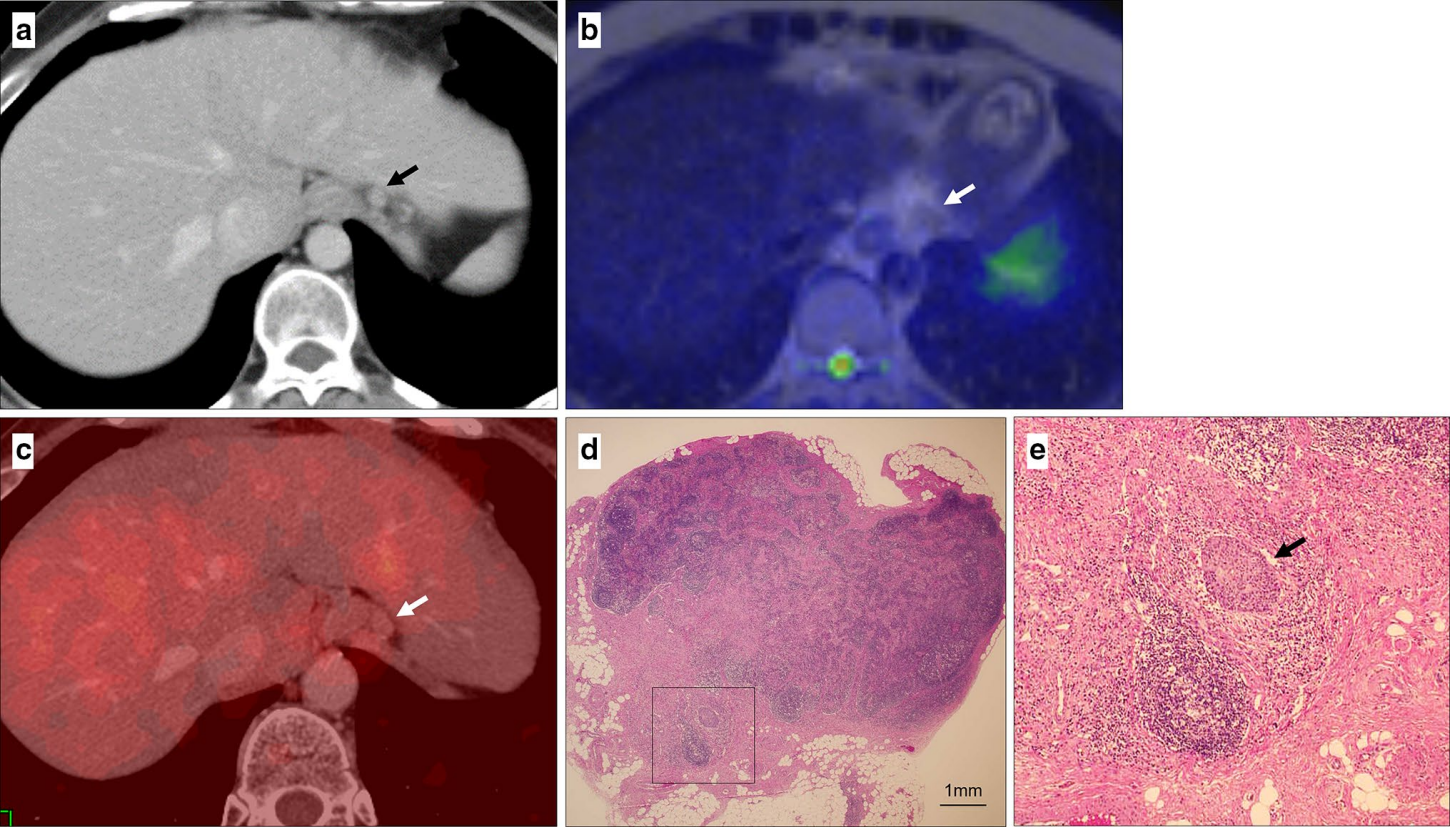
False negative lymph node of 10 mm in size by DWI and PET (arrow). **a** Paracardial lymph node was detected on contrast-enhanced CT as metastasis. It was hypointense on DWI (**b**) and hypoaccumulate on PET (**c**). **d**, **e** The node was identified as metastatic with micrometastasis (**d** square, **e** arrow)

Within 2–3 weeks prior to surgery, magnetic resonance imaging (MRI) was performed with a 1.5 T body scanner equipped with a phased array body coil (Achiva 1.5 T Nova Dual; Philips Medical Systems, Best, Heeren, Netherlands). A single-shot spin-echo type of echo-planar sequence was used to obtain diffusion-weighted magnetic resonance images. The fat signals were suppressed using short-tau inversion recovery. The *b* values corresponding to diffusion-sensitizing gradients were 0 and 1000 s/mm^2^. Sequential sampling of the k-space was used with an effective echo time (TE) and an acquisition matrix of 160 × 125, which was interpolated to 256 × 256 during image calculation. Repetition time (TR) and TE were 7800 ms and 65 ms, respectively. Slices from the cricoid cartilage to the upper abdomen were acquired with a 400-mm field of view, a 4-mm slice thickness, and a -1-mm slice gap. T2-weighted images were obtained with the following parameters: TR/TE 1000/110, train length of 15, acquisition of four signals, 256 × 204 matrices, 32-cm field of view, and 4-mm section thickness. Imaging data were transferred to an image-processing workstation (Aze Virtual Place Advanced Plus, Aze, Tokyo, Japan). A hyperintensity node with a minimum apparent diffusion coefficient value (ADC) of < 1.2 s/mm^2^ on T2- and DWI-fused images was diagnosed as positive for metastasis (Fig. 1), because the tissue diffusion level of ACD > 1.2 is not distinct (Fig. 2). The largest node was used for evaluation if two or more positive nodes were involved in one nodal station.

### FDG-PET imaging and analysis

Within 2–3 weeks prior to surgery, PET was performed using a single whole-body PET system (Advance NXi, GE Medical Systems, Milwaukee, WI, USA). After an injection of 10 mCi of FDG tracer, scanning was initiated from the top of the brain to the upper thigh with a rotating external source by the simultaneous 4-min emission and 2-min transmission method. Attenuation-corrected transaxial images were reconstructed by the ordered subsets expectation maximization algorithm into 128 × 128 matrices, a 55-cm field of view, and a 4.25-mm section thickness. Prior to PET examination, non-enhanced CT scans were performed with a 64-row multidetector CT scanner (LightSpeed VCT; GE Medical Systems, Milwaukee, WI, USA) with a 1.375-mm pitch, a 320-mm field of view, and a 1-mm thickness of reconstruction. PET and CT images were transferred to an image-processing workstation (eNTEGRA, GE Medical Systems, Milwaukee, WI, USA). A hyperaccumulation node with a maximum standardized uptake value of > 3.0 was defined as positive on PET- and CT-fused images, because the FDG uptake level of SUV < 3.0 is obscure. The largest node was used for evaluation if two or more positive nodes were contained in one nodal station. The blood sugar levels of all patients were less than 140 mg/dL at the time of PET scanning.

### Postoperative follow-up

Patients were followed up every 3–6 months for the first 5 years, and then on an annual basis. Contrast-enhanced CT scans of the neck, chest, and abdomen were performed 6 months and endoscopy was performed yearly. Patients with recurrent disease received first-line chemotherapy consisting of 5-fluorouracil and cisplatin and, if possible, additional radiotherapy.

### Statistical analysis

Statistical significance was evaluated by the Mann–Whitney *U* test for comparison of two continuous parameters and the Chi-square test for categorical variables. As regards diagnostic abilities, the web-based statistical calculator was used for analysis (two-way contingency table analysis, https://statpages.info/ctab2x2.html) [21]. Survival curves were plotted by the Kaplan–Meier method and the differences were evaluated by the log-rank test. A Cox proportional hazard regression model was used to analyze prognostic factors by univariate and multivariate analyses. A multivariate analysis was carried out using the risk factors with *P* values of < 0.1 according to the univariate analysis. Two-tailed *P* values of < 0.05 were considered significant. All statistical analyses were undertaken using SPSS v.24.0 (IBM Corp., Armonk, NY, USA).

## Results

### Metastatic lymph node status

A total of 3686 LNs containing 123 histologically proven metastatic LNs were surgically harvested. These nodes were interpreted as 1229 node stations with 94 metastases. Abdominal metastatic nodes around the stomach (*n* = 43, 45.7%) were most frequently observed. The median size of metastatic nodes and intranodal cancer nests were 7.0 mm and 3.25 mm, respectively. Thirty-six (38.3%) LNs had an intranodal cancer nest in a small area, which was the second most frequent (Table 2).

**Table 2:** Characteristics of pathological metastatic lymph node

Characteristics	Total, *n* = 94
Location, *n* (%)	
Cervical	14 (14.9)
Upper thoracic	19 (20.2)
Middle thoracic	14 (14.9)
Lower thoracic	4 (4.3)
Abdominal	43 (45.7)
Size, mm	
Whole lymph node	7.0 (3–28)
Intranodal cancer nest	3.25 (0.1–28)
Occupied area of intranodal cancer nest, *n* (%)	
< 1/3	36 (38.3)
1/3 ≤ < 2/3	17 (18.1)
2/3≤	41 (43.6)

Continuous data are shown as median (range)

### Diagnostic ability for LN evaluation

In a patient-by-patient evaluation to compare cN stage with pN, patients with clinical or pathological supraclavicular LN metastasis were excluded (*n* = 5), because they were classified as M1 disease. In a total of 71 patients, the accuracies of N staging were statistically similar between DWI and PET (68% [48/71] vs. 62% [44/71], *P *= 0.489). DWI had a tendency towards overestimation (11% [8/71] vs. 3% [2/71], *P *= 0.097) and PET towards underestimation (21% [15/71] vs. 35% [25/71], *P *= 0.092) (Table 3).

**Table 3:** Comparison of clinical N stage and pathological N stage

Pathological N stage	Number of patients	DWI-cN				PET-cN			
		cN0	cN1	cN2	cN3	cN0	cN1	cN2	cN3
pN0	37	31	6	0	0	35	2	0	0
pN1	20	6	12	2	0	13	7	0	0
pN2	13	1	7	5	0	6	5	2	0
pN3	1	0	1	0	0	1	0	0	0

Patients with clinical or pathological supraclavicular lymph node metastasis were excluded

Accuracy: DWI, 68%; PET, 62%. Overestimation: DWI, 11%; PET, 3%. Underestimation: DWI, 21%; PET, 35%

*DWI*-*cN* Clinical N stage by DWI, *PET*-*cN* Clinical N stage by PET

In a station-by-station evaluation, DWI showed higher sensitivity (67% vs. 32%, *P *< 0.001), higher negative predictive value (97% vs. 95%, *P *= 0.001), and lower specificity (98% vs. 99%, *P *= 0.010) (Table 4).

**Table 4:** Diagnostic ability of station-by-station evaluation

Diagnostic ability	DWI	PET	*P* value
Sensitivity	67 (63/94)	32 (30/94)	< 0.001
Specificity	98 (1117/1135)	99 (1130/1135)	0.010
Positive predictive value	78 (63/81)	86 (30/35)	0.448
Negative predictive value	97 (1117/1148)	95 (1130/1194)	0.001
Accuracy	96 (1180/1229)	94 (1160/1229)	0.073

Data are shown as percentage (number of proportion). *P* values were analyzed by the web-based calculator at StatPages (two-way contingency table analysis, https://statpages.info/ctab2x2.html)

*DWI* diffusion-weighted magnetic resonance image, *PET* positron emission tomography, *N*+ positive for metastasis

### Sensitivities by station-by-station evaluation relating to nodal status

DWI had higher sensitivity for LN location in the cervical and upper thoracic area, middle- and large-sized LNs, and intranodal cancer nest sizes of all categories. Despite the same sensitivity for small-sized nodes (25% vs. 25%), DWI showed higher sensitivity for small-sized cancer nests (53% vs. 23%, *P *< 0.001), particularly, DWI exhibited 100% sensitivity for large-sized cancer nests. As the occupied area of cancer nests increased, sensitivities also significantly increased in DWI (*P *< 0.001), which was not observed in PET (Table 5).

**Table 5:** Sensitivities by station-by-station evaluation relating to nodal status

Metastatic lymph node status	DWI, %	PET, %	*P* value
Node location			
Cervical	86 (12/14)	14 (2/14)	< 0.001
Upper thoracic	68 (13/19)	26 (5/19)	0.022
Middle-lower thoracic	67 (12/18)	33 (6/18)	0.056
Abdominal	60 (26/43)	40 (17/43)	0.084
Node size			
Small size, < 5 mm	25 (3/12)^a^	25 (3/12)^d^	1.000
Middle size, ≥ 5 to < 10 mm	61 (28/46)^a^	22 (10/46)^d^	< 0.001
Large size, > 10 mm	89 (32/36)^a^	47 (17/36)^d^	< 0.001
Intranodal cancer nest size			
Small size, < 5 mm	53 (33/62)^b^	23 (14/62)^e^	0.001
Middle size, ≥ 5 to < 10 mm	89 (16/18)^b^	33 (6/18)^e^	0.002
Large size, > 10 mm	100 (14/14)^b^	71 (10/14)^e^	0.049
Occupied area of intranodal cancer nest			
Small area, < 1/3	42 (15/36)^c^	25 (9/36)	0.145
Middle area, ≥ 1/3 to < 2/3	76 (13/17)^c^	29 (5/17)	0.015
Large area, ≥ 2/3	85 (35/41)^c^	39 (16/41)	< 0.001

Data are shown as percentage (number of lymph nodes)

*DWI* diffusion-weighted magnetic resonance image, *PET* positron emission tomography

^a,b,c^*P* < 0.001

^d^*P* = 0.042

^e^*P* = 0.002

### True positive node and false negative node

**Fig. 3:**
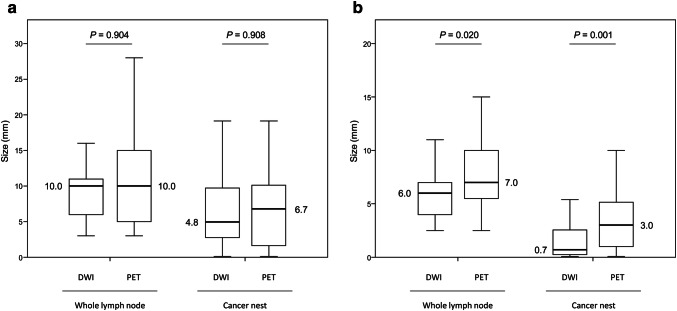
Comparison of the size of true positive nodes and false negative nodes between DWI and PET. **a** True positive node. **b** False negative node

True positive (TP) LNs and false negative (FN) LNs were compared in relation to the whole LN size and the cancer nest size. Regarding TP LNs, both sizes were similar between both modalities, whereas in relation to FN LNs, both sizes were significantly smaller in DWI (whole node, 6.0 mm vs. 7.0 mm, *P *= 0.020; cancer nest, 0.7 mm vs. 3.0 mm, *P *= 0.001, respectively) (Fig. 3).

### DWI-N status and postoperative survival

**Fig. 4:**
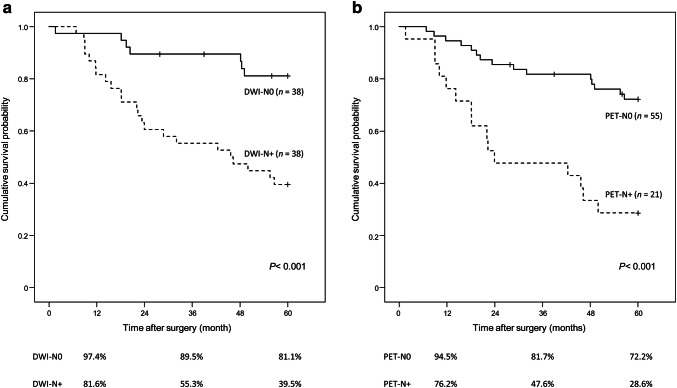
Overall survival curves according to DWI-N status and PET-N status. **a** DWI-N status. **b** PET-N status. DWI-N0, clinical negative for lymph node metastasis by DWI; DWI-N+, clinical positive for metastasis by DWI; PET-N0, clinical negative for lymph node metastasis by PET; PET-N+, clinical positive for lymph node metastasis by PET

**Fig. 5:**
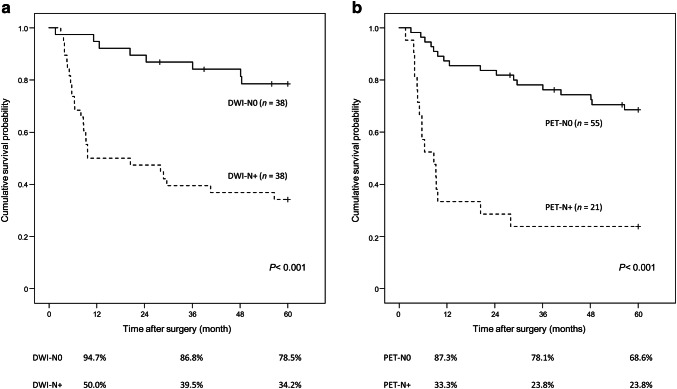
Relapse-free survival curves according to DWI-N status and PET-N status. **a** DWI-N status. **b** PET-N status. *DWI-N0* clinical negative for lymph node metastasis by DWI, *DWI-N+* clinical positive for metastasis by DWI, *PET-N0* clinical negative for lymph node metastasis by PET, *PET-N+* clinical positive for lymph node metastasis by PET

The clinical positive for LN metastasis by DWI (DWI-N+) group had significantly more pathological metastatic LNs than the clinical negative for LN metastasis by DWI (DWI-N0) group (3.00 ± 3.11 vs. 0.24 ± 0.59, *P *< 0.001). Similarly, pathological metastatic nodes were more often identified in the clinical positive for LN metastasis by PET (PET-N+) group than in the clinical negative for LN metastasis by PET (PET-N0) group (3.48 ± 3.69 vs. 0.91 ± 1.63, *P *< 0.001). The DWI-N+ group demonstrated a significantly lower 5-year overall survival (OS) than the DWI-N0 group (39.5% vs. 81.1%, *P *< 0.001), as did the PET-N+ group (Fig. 4). Likewise, the DWI-N+ group had a worse 5-year relapse-free survival rate (RFS) than DWI-N0 (34.2% vs. 78.5%, *P *< 0.001), as did the PET-N+ group (Fig. 5; Table 6).

**Table 6:** Risk factors for overall survival by univariate and multivariate analysis

Clinical risk factor	Category	Univariate analysis			Multivariate analysis		
		Hazard ratio	95% CI	*P* value	Hazard ratio	95% CI	*P* value
Age,	< 67	1.202	0.586–2.464	0.615			
	≥ 67						
Gender	Female	1.584	0.679–3.968	0.287			
	Male						
Tumor location	Upper/middle	1.171	0.564–2.432	0.673			
	Lower/abdominal						
Histological grade	G3	1.102	0.384–3.157	0.857			
	G1/G2						
Lymphadenectomy	2-field	1.195	0.488–2.924	0.696			
	3-field						
Clinical T stage	T3/T4a	5.393	2.570–11.316	< 0.001			
	T1/T2						
Clinical N stage	N2/N3	5.458	2.373–12.555	< 0.001			
	N0/N1						
Clinical M stage	M1	3.084	0.730–13.035	0.126			
	M0						
Clinical stage	III/IV	3.782	1.836–7.791	< 0.001	1.676	0.701–4.008	0.246
	I/II						
DWI-N status	DWI-N+	4.413	1.889–10.310	0.001	2.522	0.905–7.030	0.077
	DWI-N0						
PET-N status	PET-N+	3.957	1.921–8.154	< 0.001	1.904	0.793–4.571	0.150
	PET-N0						

*95% CI* 95% confidence interval, *DWI* diffusion-weighted magnetic resonance image, *PET* positron emission tomography, *Upper, middle and lower* location of thoracic esophagus, *DWI*-*N*+ positive for metastasis by DWI, *PET*-*N*+ positive for metastasis by PET

### Prognostic significance of DWI-N and PET-N status

The correlation between OS/RFS and clinical risk factors was assessed by univariate and multivariate analyses. For cancer status, because cT, cN and cStage factors showed similar significant *P* values, cStage, which reflected cT and cN status, was adopted as a covariate. As a result, although no significant risk factors were identified for 5y-OS in multivariate analysis, DWI-N status had a tendency toward an independent risk factor (*P *= 0.077) (Table 6). Meanwhile, for 5y-RFS, DWI-N status was an independent prognostic factor (hazard ratio [HR], 2.642; *P *= 0.048), as was PET-N status (HR, 2.418; *P *= 0.033) (Table 7).

**Table 7:** Risk factors for relapse-free survival by univariate and multivariate analysis

Clinical risk factor	Category	Univariate analysis			Multivariate analysis		
		Hazard ratio	95% CI	*P* value	Hazard ratio	95% CI	*P* value
Age	< 67	1.605	0.798–3.229	0.185			
	≥ 67						
Gender	Female	1.427	0.619–3.290	0.404			
	Male						
Tumor location	Upper/middle	1.008	0.506–2.012	0.981			
	Lower/abdominal						
Histological grade	G3	1.056	0.408–2.738	0.910			
	G1/G2						
Lymphadenectomy	2-field	1.135	0.492–2.616	0.766			
	3-field						
Clinical T stage	T3/T4a	5.101	2.515–10.348	< 0.001			
	T1/T2						
Clinical N stage	N2/N3	4.575	2.022–10.353	< 0.001			
	N0/N1						
Clinical M stage	M1	5.768	1.304–25.506	0.021			
	M0						
Clinical stage	III/IV	3.845	1.920–7.702	< 0.001	1.596	0.709–3.591	0.259
	I/II						
DWI-N status	DWI-N+	4.786	2.149–10.660	< 0.001	2.642	1.010–6.913	0.048
	DWI-N0						
PET-N status	PET-N+	4.778	2.384–9.577	< 0.001	2.418	1.076–5.436	0.033
	PET-N0						

*95% CI* 95% confidence interval,*DWI* diffusion-weighted magnetic resonance image, *PET* positron emission tomography, *Upper, middle and lower* location of thoracic esophagus, *N *+ positive for metastasis

## Discussion

In this study, we clarified the diagnostic performance of DWI for assessing LN metastasis in patients with eSCC compared with FDG-PET. In a station-by-station analysis, the sensitivity of DWI was better than that of PET. The sensitivity of PET in the present study was consistent with that of previous studies [8, 9, 22, 23]. Although there were also statistical differences in specificity and negative predictive value, we consider that these differences do not have a potential impact on clinical practice, because each value was almost equal between both modalities with more than 90% probability by a large number of non-metastatic nodes. Therefore, we focused on sensitivity. Consequently, DWI was a little better or equal to CT in sensitivity [24, 25], which seemed not to reach a satisfactory result. Meanwhile, in a subgroup analysis of metastatic nodal status, DWI showed better sensitivity in cervical and upper thoracic LNs. We believe that the reason for this is that cervical LNs were sensitive in DWI, because they exist near the body surface, and cervical/upper thoracic LNs might have been overlooked as inflammatory LNs during PET image interpretation. DWI exhibited high performance with more than 85% sensitivity for large nodes, middle- and large-sized cancer nests, and large cancer nest areas in the whole node, better than PET. In parallel with occupied areas of growing cancer nests, the sensitivities increased in DWI, which was not observed in PET. In addition, FN LNs of DWI were smaller in whole node size and in cancer nest size. We consider DWI may be affected more by the cancer cell volume for detecting metastatic LNs compared with PET.

As a functional imaging technique, PET was able to sensitively reflect metabolic changes in tissue and showed higher diagnostic sensitivity for the earlier occurrence of metabolic rather than morphological changes in tissue lesions compared with CT [26, 27]. From our above-mentioned results, we additionally speculate that LNs may change through the three phases of metastasis. First, tumor cell density may change followed by elevation of intranodal pressure, then nodal metabolic alternations occur corresponding to cancer cell increases, and finally morphological LN changes such as shape, contrast-enhanced differentiation, and increased size ensue. DWI, which depends upon cellular density and interstitial pressure, may reflect these changes in earlier phases of cancer progression during the process of intranodal metastasis.

Because eSCC frequently metastasizes to small nodes in early stage disease, discrimination of metastatic disease from non-metastatic has been considered difficult [28, 29]. Conventional EUS and recent EUS elastography have greater sensitivity in nodal detection [6, 7, 25], but have disadvantages in evaluating nodes distal from the esophageal wall and stenotic tumors and depend on the investigator’s skill and the diagnostic criteria. Although DWI has more limitations in time and spatial resolution and anatomical information than CT, and disadvantages in evaluating distal metastasis compared with PET, its advantages include no radiation exposure and no allergic reaction to the contrast agent, and it is less costly, easy to use, and non-invasive. In association with MRI, ultra-small superparamagnetic iron oxide particle (Ferumoxtran-10)-enhanced MRI lymphangiography has the highest performance for detecting nodal metastasis of esophageal cancer with an overall sensitivity of 88–100% in small initial studies [30, 31]; however, its clinical use has not yet been approved in Japan. We expect the future clinical utility of DWI for these reasons.

In this study, PET-N+ patients had greater numbers of pathological metastatic nodes than PET-N0, consequently, PET-N+ patients showed a worse 5y-OS and 5y-RFS, similar to previous reports [23, 32]. Additionally, in this study we revealed DWI-N+ patients also had more pathological metastatic nodes and a worse prognosis than DWI-N0, as well as PET-N status. We believe preoperative DWI-N status may identify high-risk populations for postoperative courses, also similar to PET. Although the multivariate analysis for OS was not significant, DWI-N status was expected to be a potential risk factor. More sample volume is needed to confirm this point. Meanwhile, for RFS, DWI-N status was identified to be an independent prognostic factor, as was PET-N status. Clinical stage was mainly based on CT findings in this study. The sensitivity of DWI was nearly equal to CT, which seemed unsatisfactory; however, DWI-N status reflected postoperative outcome, especially in RFS, suggesting that DWI may have a significant performance in clinical practice. We consider DWI is not only a modality of LN diagnosis but also a predictive modality after eSCC surgery. In other words, DWI-N+ patients require preoperative neoadjuvant chemotherapy and, if possible, may be recommended postoperative adjuvant chemotherapy.

There were several limitations of our study. PET evaluation was performed using conventional PET equipment. If integrated PET/CT was applied, we suppose, the findings would differ from the current results. Moreover, in eSCC, the diagnostic ability of LN metastasis by radiological modalities may reach a clinical limit because of the presence of micrometastasis in LN. Further improvement of sensitivity for LN metastasis could not be expected with already-existing radiological modalities.

In conclusion, DWI, which depends on cancer cell volume followed by elevated intranodal density, shows higher sensitivity than PET. It has clinical impact in predicting postoperative survival for patients with eSCC alongside its diagnostic ability and has significant performance in clinical practice.

## Abbreviations

LN: Lymph node
eSCC: Esophageal squamous cell cancer
CT: Computed tomography
EUS: Endoscopic ultrasonography
FDG: 18F-fluorodeoxyglucose
PET: Positron emission tomography
MRI: Magnetic resonance image
DWI: Diffusion-weighted magnetic resonance image
TR: Repetition time
TE: Echo time
cN+: Clinical positive for lymph node metastasis
pN+: Pathological positive for lymph node metastasis
DWI-N+: Clinical positive for lymph node metastasis by DWI
DWI-N0: Clinical negative for lymph node metastasis by DWI
DWI-cN: Clinical N stage by DWI
PET-N+: Clinical positive for lymph node metastasis by PET
PET-N0: Clinical negative for lymph node metastasis by PET
PET-cN: Clinical N stage by PET
TP: True positive
FN: False negative
